# FRAMEWORK FOR SYSTEMATIC IDENTIFICATION OF ETHICAL ASPECTS OF HEALTHCARE TECHNOLOGIES: THE SBU APPROACH

**DOI:** 10.1017/S0266462315000264

**Published:** 2015

**Authors:** Emelie Heintz, Laura Lintamo, Monica Hultcrantz, Stella Jacobson, Ragnar Levi, Christian Munthe, Sofia Tranæus, Pernilla Östlund, Lars Sandman

**Affiliations:** Swedish Council on Health Technology Assessment (SBU); Center for Medical Technology Assessment (CMT), Department of Medical and Health Sciences, Linköping Universityemelie.heintz@sbu.se; Swedish Council on Health Technology Assessment (SBU); Swedish Council on Health Technology Assessment (SBU); Department of Learning, Informatics, Management and Ethics (LIME), Karolinska Institutet; Swedish Council on Health Technology Assessment (SBU); Department of Philosophy, Linguistics and Theory of Science, University of Gothenburg; Swedish Council on Health Technology Assessment (SBU); Faculty of Odontology, Malmö University; Department of Dental Medicine, Karolinska Institutet; Swedish Council on Health Technology Assessment (SBU); Faculty of Odontology, Malmö University; School of Health Sciences, University of Borås; National Centre for Priority Setting in Health Care, Linköping University

**Keywords:** Ethics, Guideline, Framework, Procedure, Health technology assessment

## Abstract

**Objectives:** Assessment of ethical aspects of a technology is an important component of health technology assessment (HTA). Nevertheless, how the implementation of ethical assessment in HTA is to be organized and adapted to specific regulatory and organizational settings remains unclear. The objective of this study is to present a framework for systematic identification of ethical aspects of health technologies. Furthermore, the process of developing and adapting the framework to a specific setting is described.

**Methods:** The framework was developed based on an inventory of existing approaches to identification and assessment of ethical aspects in HTA. In addition, the framework was adapted to the Swedish legal and organizational healthcare context, to the role of the HTA agency and to the use of non-ethicists. The framework was reviewed by a group of ethicists working in the field as well as by a wider set of interested parties including industry, interest groups, and other potential users.

**Results:** The framework consists of twelve items with sub-questions, short explanations, and a concluding overall summary. The items are organized into four different themes: the effects of the intervention on health, its compatibility with ethical norms, structural factors with ethical implications, and long term ethical consequences of using the intervention.

**Conclusions:** In this study, a framework for identifying ethical aspects of health technologies is proposed. The general considerations and methodological approach to this venture will hopefully inspire and present important insights to organizations in other national contexts interested in making similar adaptations.

Health care is founded on and permeated by ethical values and norms. Hence, all use of health technology is likely to raise ethical issues of some kind. To guide decisions on the use or non-use of specific health technologies, health technology assessment (HTA) is a tool for assessing technologies from a medical, economic, social, legal, and ethical perspective (1). Hofmann's checklist of thirty-three ethics questions provided a starting point for systematic integration of ethics into HTA (2) and laid a basis for developing similar sets of questions at both national and international levels (e.g., Burls et al., 2011 [3], Saarni et al., 2011 [4], and Assasi et al., 2014 [5]). Nevertheless, how the implementation of ethical assessment in HTA is to be organized remains unclear. The existing sets of questions are also of a general nature and may need to be adjusted for use in specific regulative and organizational settings (6).

Ethical aspects are included as a vital part of the HTA reports produced by The Swedish Council on Health Technology Assessment (SBU). The assessments of ethical aspects have been based on well-established areas of concern in healthcare ethics, such as the four basic principles of medical ethics: beneficence, non-maleficence, respect for autonomy and justice (7;8). Existing checklists (2;9;10) and models (11) have also been used. However, there has been a demand for a common framework that could guide the ethical assessments in all of the HTA projects at SBU. There has also been a demand for a framework for ethical assessment adapted to effective health policy and regulation. Besides being of use at SBU, such a framework could be an important tool for other organizations or decision makers involved in quality assessment and priority-setting of Swedish health care. The need for such a common framework was emphasized in 2010 when the Swedish Health and Medical Services Act (HMSA) introduced a requirement that all new health technologies of potential importance for human value and integrity should be assessed in terms of “individual as well as social ethical aspects” (12). A follow-up in 2013, highlighted that the health care providers needed guidance on how to achieve such an implementation (13).

In this study, we present a framework for systematic identification of relevant ethical aspects of healthcare technologies. Furthermore, we describe the process of developing and adapting the framework to the specific setting in which it will be used. This work may be used by other organizations or inspire and inform others to conduct similar adaptations to their organizational or national settings.

## METHODS

### Developing the Framework

**Figure 1:**
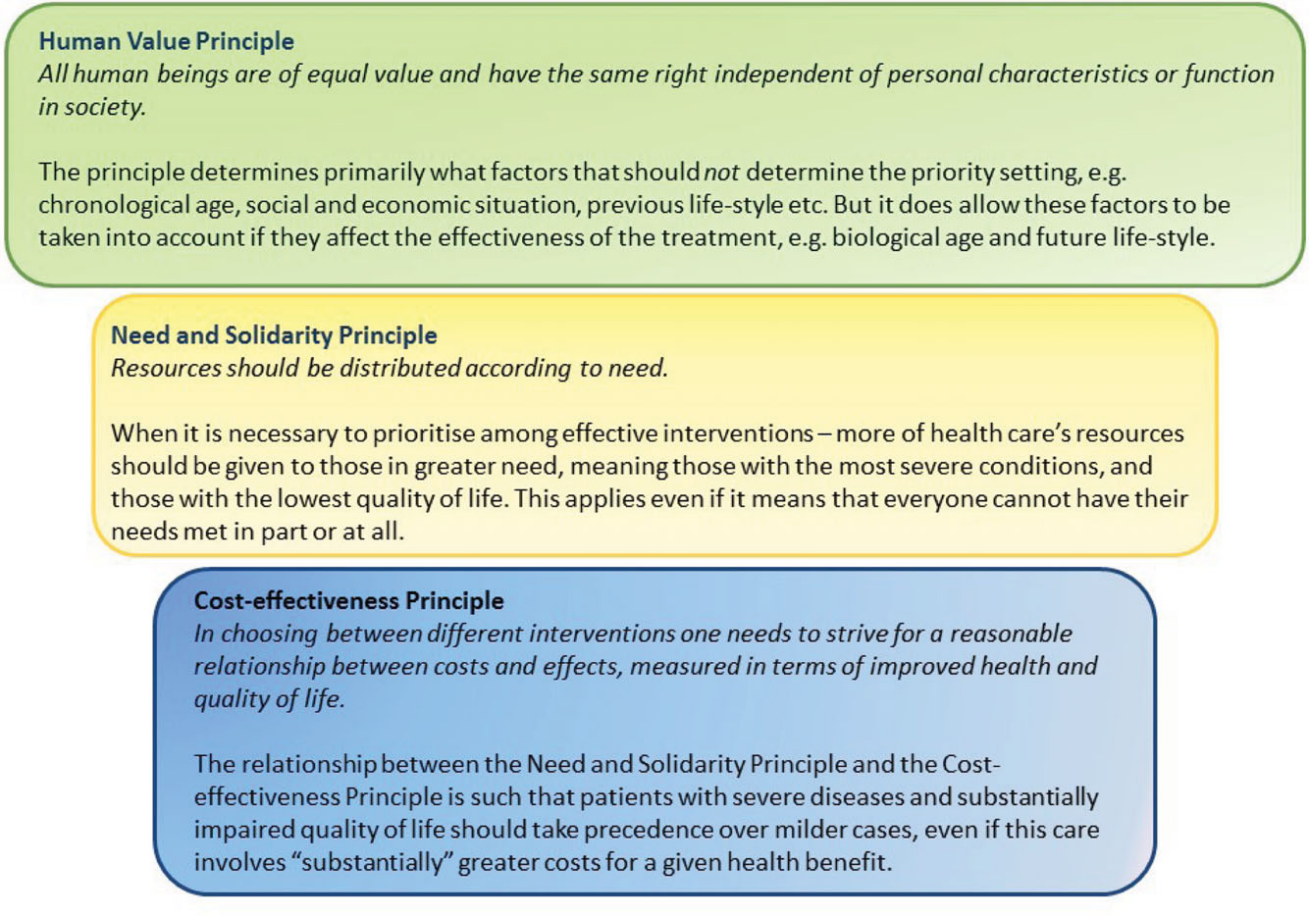
The Swedish ethical platform for priority-setting in health care (15).

An attempt to form a context-specific framework was initially made by a regional HTA agency in Sweden (14), in which the checklist by Hoffmann (2005) was adapted to the principles of the Swedish ethical platform for healthcare priority-setting (see Figure 1) (15) and other relevant features of the Swedish health-care legislation. This pilot framework was well received and rendered support for the idea of developing a version for wider application. At the same time, it highlighted the need to consider further aspects and circumstances in the development of such a framework.

To develop such a framework for wider application, a group consisting of seven HTA experts at SBU and two ethicists (henceforth called the working group) was formed and met regularly from November 2012 to May 2014. The regional checklist acted as the starting point (14). In addition, the working group identified various aspects considered important to take into account when creating a framework for ethics in HTA that is adjusted to specific healthcare contexts (see next section).

To identify other possible checklists for identification of ethical aspects in HTA, Web sites of different HTA organizations were explored. The identified checklists (2;9;10) and their references were reviewed and compared with the regional framework, resulting in some questions being added. Swedish healthcare regulation was also analyzed more in-depth, leading to further revision of the content and the structure of the framework. The following aspects of Swedish healthcare legislation were incorporated into the framework: the goal of care in terms of health (HMSA), the principles of the Swedish ethical platform for priority setting (included in HMSA), autonomy (HMSA), and privacy (HMSA and Patient Data Act, PDA).

To validate the first draft of the framework, it was sent for review to six academic ethicists and one health economist with special training in ethics. The ethicists represented both theoretical and empirical research in the areas of medical ethics, practical philosophy and nursing science. Based on the feedback received, the framework was modified further. The comments helped to clarify the questions and reorganize the structure without essentially changing the content of the framework.

Further validation was performed through internal review at SBU, as well as through the involvement of other healthcare organizations, political bodies, interest groups, and parts of industry identified as potential stakeholders. These included the Swedish national council on medical ethics (SMER), the Swedish national parliament, national governmental representatives, regional HTA agencies, interest groups within the medical technology and pharmaceutical industry, insurance companies, and unions/other professional organizations. This further round of review resulted in thirty responses and led to additional revisions of the structure of the framework and clarifications of the text. These reviews did not affect the content matter of the framework.

### Aspects to Consider when Adapting General Checklists to Specific Contexts

During the project, we identified four aspects to consider when developing a framework for ethics in HTA that is adjusted to specific healthcare contexts. These have all been taken into account in the development of the framework discussed in this study.

*Stages of the HTA process:* The more general checklists do not distinguish between specific stages of the HTA process. This can lead to problems with identifying the most relevant questions for each specific stage, for example, when prioritizing between proposed project, in the scoping of projects or when assessing the technology.

*National legislation and regulation:* Laws, regulations, and case rulings of agencies or courts in the specific healthcare contexts may express different standpoints on ethical issues. These standpoints may provide guidance for assessments of ethical aspects performed in particular contexts. For instance, legislative texts may provide guidance on how to address issues concerning equity, autonomy, and privacy.

*Organization and funding of health care:* Organization and funding of the healthcare system may influence what is relevant to include in the framework. Some questions to consider are: (i) How is the healthcare technology funded in the particular context and does this have implications for what interests and organizational arrangements that need to be taken into account? In particular, what possible conflicts of interest may need to be accounted for? (ii) What is the role of the organization that will be using the framework? Is it to issue recommendations or to make an assessment that will be used by other stakeholders? (iii) Does the framework need to be adapted to existing working processes within the agencies and/or organizations that will use the framework?

*Available ethical expertise:* Assessment of ethical aspects requires insight into ethical theory and argumentation. For the framework to be more useful for users with limited ethics training, explanations and examples may be necessary. In addition, this means that when using the framework one ought to consider if, when, and how specific ethics expertise is to be involved.

## RESULTS

Three different versions of the ethics framework were considered necessary: one for prioritization and scoping of HTA projects, one for identification of ethical aspects of the specific technologies being assessed, and finally, one for prioritization of research questions. In this study, only the framework for identification of ethical aspects when assessing health technologies is presented. This section begins with a description of this framework. Next, specific adaptations of this framework to healthcare legislation, and organization of the healthcare system, as well as available ethical competence, are described. Finally, a process for using the framework is proposed.

### Outline of the Framework's Content and Structure

**Figure 2:**
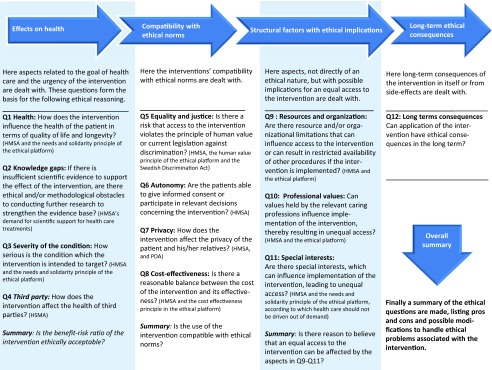
Overview of the structure of argumentative priority of the framework assessment as a basis for such decisions. HMSA: Swedish Health and Medical Services Act, PDA: Patient Data Act.

**Figure 3:**
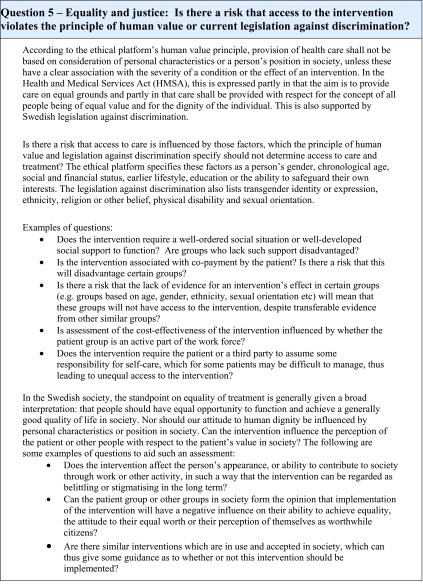
Example of a question in the framework for identification of ethical aspects for assessment of health technologies.

The framework consists of twelve items with sub-questions and short explanations (see Figure 2 for an overview, and Figure 3 for an example of one of the items), as well as an overall concluding summary. The items are organized into four different themes: the effects of the intervention on health, its compatibility with ethical norms, structural factors with ethical implications, and long-term ethical consequences of using the intervention. The structural order of the questions, illustrated by the figure below, reflects an *argumentative priority* of the items.

The first theme concerns the intervention's effect on patient health, including whether there are ethical or methodological problems in substantiating that effect, the severity of the condition, as well as health effects on third parties. If a technology exhibits no, too weak, or too badly substantiated positive benefits over risk ratio, there is no need to consider the other themes to be able to conclude that the technology should not be used. If the technology is currently being used, this ratio will form an important background for the assessment of the other themes of the framework. The final assessment within this theme relates to the balance between benefits and harms given the aim of the health technology, the urgency of the intervention, given the severity of the condition, and to what extent lack of evidence due to ethical or methodological factors may be tolerated.

The next theme, compatibility with ethical norms, covers aspects of equality and justice, autonomy, privacy, and cost-effectiveness. If any of these are violated to an unacceptable degree, further pondering of the other themes will not be able to change the fact that the technology is unacceptable. If it has some ethical downsides of less serious kinds, these will form an important background to the assessment within the subsequent themes.

The theme on structural factors contains questions on how resources, organizations, professional values and roles, as well as stakeholder interests, affect equal access to health care. The theme on long-term ethical consequences complements the others to ensure a more inclusive scope than immediate clinical or societal concerns.

The final item is a summary of all of the ethical aspects raised by the technology. This summary is not by itself a conclusion. Instead, it is thought to describe issues to ponder further, arguments for and against the use or the implementation of the technology, as well as suggested modifications of the technology in response to such ethical concerns. To the extent the ethical norms in Swedish legislation do not give specific guidance, these arguments might voice different ethical perspectives on the identified aspects.

### A Proposed Process for Using the Framework

Successful use of the framework requires integration with a specific working process that suits the existing administrative circumstances and the jurisdictional context. This is necessary to maximize the usefulness of the framework for specific HTA agencies and healthcare decision makers. It is also necessary to identify a proper place and form for involving ethical expertise in HTAs (16). Here, we present an example of how the framework may be used in a HTA process by using the case of SBU.

First, the project group is recommended to start to reflect on potential ethical aspects of the assessed technology *without* the use of the framework (at SBU the project group consists of internal staff members and external field experts). This initial “brain-storming” exercise is done to not direct the focus of attention away from issues that spontaneously are viewed as ethically challenging within the field. The brainstorming exercise is led by the project director, but the group members are basically free to voice any ethical problems that they associate with the technology. The result is documented and later assessed in relation to the outcome of the more systematic approach using the framework. Second, the project group systematically uses the framework to identify further possible ethical challenges. A possible consequence of these two steps is the need to modify the scope of the project, which could lead to further literature searches on the topic as whole, or on specific ethical issues (17). Third, as the assessment of the scientific evidence proceeds, with the possible addition of new search results, the project group may need to return to the framework for further reflection on whether the evidence modifies or deepens the initial ethical assessment. If more substantial and difficult ethical issues are identified during this process, the project group is recommended to consult professional ethics expertise for support or for a more full-fledged ethical analysis of the assessed technology. At this point, a systematic approach for such expert involvement has not yet been formally developed at SBU. However, the group is recommended to liaise with a government advisory body on medical ethics (The Swedish National Council on Medical Ethics) if the technology raises, what appears to be new ethical issues, or issues of general importance from a societal perspective. Thus, the decision concerning the involvement of ethical expertise is currently in the hands of the ones conducting the HTA. Finally, the result of the assessment is summarized as a list of ethical pros and cons of the technology. This is done even if some of the themes on the list rules out the intervention, as there is always a need to clarify the entire argumentative situation, in case other parties assess it differently.

## DISCUSSION

We have presented the process and the main results of the development of a framework for identifying ethical aspects in the assessment of health technologies. This framework will be used by SBU, but other Swedish healthcare organizations have also shown an interest. The framework meets the need of a systematic approach to identify ethical aspects for further analysis in the context of HTA. In Sweden, this need has recently been emphasized by a legal requirement on ethical assessment of all new healthcare technologies (12), resulting in healthcare providers requesting guidance on how to perform such assessment (13). The study describes how the framework has been adapted to suit the specific context of HTA and Swedish health care. In this adaptation, several factors to bear in mind when adapting and complementing previously available checklists to specific contexts have been identified. Hopefully, other jurisdictions and healthcare policy contexts beside HTA can learn from this process and its outcome.

In particular, the framework as a whole possesses four specific qualities:

(i) The presented framework is adapted to one specific part of the HTA process, that is, the actual assessment of technologies. However, because ethical queries are raised in other parts of the process, for example, prioritization of project proposals or prioritization of research proposals to fill established knowledge gaps, this framework will be part of a more comprehensive set of frameworks. This allows for a refinement of the ethical questions needed to be raised at each specific stage that would not be available with only one framework for the entire HTA process.
(ii) As the framework has been developed for identifying ethical aspects of the assessed technology, and not to perform a full-fledged ethical analysis, it may also serve as a tool for the project group to decide if and when further expertise in ethics needs to be consulted. It may further serve as a bridge between the views of the project group and the involved ethical expertise concerning the role of ethics in HTA.
(iii) It is structured into a logical order of argumentative priority, emphasizing more clearly than existing checklists how the questions are linked to each other and how the answers to some of the questions depend upon the answers to previous questions (see Figure 2).
(iv) It has been adapted to the specific jurisdictional and healthcare policy context as well as to the HTA process. Hence, the framework provides both guidance and consistency in how to approach ethical assessment in relation to current laws and regulations of the context.

Reviewers consulted in the process of designing the framework agreed that it captures the ethical standpoints to address in Swedish health care, although some of them remarked that some questions go beyond strictly clinical ethics, that is, ethical problems arising in the context of interacting with patients. Furthermore, some of the reviewers did not consider questions about relevant effects of the technology as strictly ethical. However, we would argue that these aspects clearly relates to important ethical norms in form of beneficence etc. This is a consequence of the attempt to capture both core clinical ethical values and norms, as well as what Hofmann refers to as process, social or interactive aspects of the technology, linked to overall effects of its use in a specific social context (18).

Some of the consulted ethicists raised concerns that the framework might trivialize the complexity of ethical analysis. Although understandable, such criticism overlooks the fact that the framework is not itself a vehicle for solving ethical issues, only for identifying and clarifying them. Besides the questions and their argumentative priorities, the framework is also presented together with a proposed process for its use, including the possibility to consult ethical expertise to assist with more in-depth analysis when needed. Of course, this suggests that users need to have sufficient knowledge of ethical assessment to be able to identify when the complexity of ethical issues require consultancy of professional ethicists. This further accentuates the need for sufficiently strong organizational support, in the form of appropriate education for professionals using the framework. In addition, any use of the framework should be associated with a clarified working process. The organization needs to specify when and how the matter of possible involvement of ethics expertise is to be raised. Finally, the proposed process of how to use the framework prescribes an open-ended start, where ethical aspects are pondered upon independently of the actual list of structured questions.

Another concern has been that the ethical assessment results in a recommendation, while the role of SBU as a HTA agency is to make an objective assessment that is directed to decision makers. Indeed, it may be difficult to draw a strict line between assessment and appraisal when it comes to normative issues (19). However, this has been taken into account by not requiring that the ethical assessment leads to a strict conclusion on whether the considered intervention is ethically acceptable or not. This suggests that the ethical assessment cannot (without qualification) be used to decide on coverage or not or whether it should be recommended or banned, etc. Rather, the result of the assessment is summarized as an informative list of pros and cons of the technology from the different ethical perspectives compatible with Swedish healthcare legislation, and it is up to the decision makers to draw a conclusion based on this information. At the same time, this opens up for a possible problem for decision makers. If lacking ethics training, it may be difficult to assess the importance of the ethics analysis in the bulk of information that they are to base their decisions upon.

## CONCLUSIONS

This study presents a framework for identifying ethical aspects of health technologies. It also illustrates how specific regulations and contexts may suggest special adaptions and variations to such a framework and the process for its use. In particular, the framework includes a wider set of issues than traditional clinical ethical concerns, otherwise at the center of healthcare ethics discourse. The general considerations and methodological approach to this venture will, we hope, inspire organizations in other national contexts that are interested in making similar adaptations and provide important insights that might inform this work.

## CONFLICTS OF INTEREST

The authors have no conflicts of interest to declare.
